# Suicide and suicide attempt in users of GLP-1 receptor agonists: a nationwide case-time-control study

**DOI:** 10.1016/j.eclinm.2024.103029

**Published:** 2024-12-31

**Authors:** Julien Bezin, Anne Bénard-Laribière, Emilie Hucteau, Marie Tournier, François Montastruc, Antoine Pariente, Jean-Luc Faillie

**Affiliations:** a University Bordeaux, INSERM, BPH, Team AHeaD, U1219, F-33000, Bordeaux, France; b CHU de Bordeaux, Clinical Pharmacology Unit, INSERM, U1219, F-33000, Bordeaux, France; c Hospital Charles Perrens, Bordeaux, France; d Department of Medical and Clinical Pharmacology, Centre of PharmacoVigilance and Pharmacoepidemiology, Faculty of Medicine, Toulouse University Hospital (CHU), Toulouse, France; e CIC 1436, Team PEPSS (Pharmacologie En Population cohorteS et biobanqueS), Toulouse University Hospital, Toulouse, France; f CHU Montpellier, Service de Pharmacologie Médicale et Toxicologie, Montpellier, France; g University Montpellier, INSERM, Institut Desbrest d’Épidémiologie et de Santé Publique, Montpellier, France

**Keywords:** Suicide and suicide attempt, Glucagon-like peptide-1 receptor agonists, Dipeptidyl peptidase-4 inhibitors, Self-controlled study, Reimbursement healthcare databases

## Abstract

**Background:**

Glucagon-like peptide-1 receptor agonists (GLP-1 RA) are extensively evaluated for the risk of suicidal behaviors or ideation; the influence of psychiatric history or obesity on this potential effect remains to be investigated. Therefore, we aimed to assess the association between GLP-1 RA and suicide or suicide attempt, considering these factors.

**Methods:**

Patients ≥18 y who died by suicide or were hospitalized for suicide attempt (2013–2021) with at least one GLP-1 RA dispensing within the 180 preceding days were selected from the French National Health Data System (SNDS). A case-time-control design compared, for each patient, GLP-1 RA exposure in the 30 days preceding the outcome (composite of suicide or suicide attempt) to three earlier 30-day reference periods. Potential exposure trend bias was controlled using up to five time-controls matched on age, sex, psychiatric history, obesity, calendar time. Analyses were adjusted for time-varying confounders. Finally dipeptidyl peptidase-4 (DPP-4) inhibitors were studied as negative controls for potential biases.

**Findings:**

This study included 1102 cases and 5494 controls. Mean case age was 57.4 years (SD 11.4); 44.6% were male, 67.6% had a recent psychiatric history and 51.3% had obesity. GLP-1 RA use was not associated with an increased risk of suicide or suicide attempt (OR, 0.62; 95% CI, 0.51–0.75), with consistent results for DPP-4 inhibitors (0.75; 0.67–0.84). Results obtained according to recent psychiatric history and obesity were comparable.

**Interpretation:**

This large nationwide case-time-control study provides reassurance about the short-term psychiatric safety of GLP-1 RA, showing no specific risk for patients with psychiatric disorders or obesity.

**Funding:**

French Medicines Agency.

**Research in context**

**Evidence before this study**

Signals of depression and/or suicide risk associated with glucagon-like peptide-1 receptor agonists (GLP-1 RA) emerging from pharmacovigilance data, which contradict meta-analysis of clinical trial data, have led to the conduct of several pharmacoepidemiological safety studies. Based on the following PubMed query, we searched for all post-marketing studies that evaluated this risk in population (“Glucagon-Like Peptide-1 Receptor Agonists” [Mesh] OR GLP-∗ OR semaglutide OR liraglutide OR exenatide OR dulaglutide OR tirzepatide OR lixisenatide OR exenatide) AND (“Suicide” [Mesh] OR “Suicide, Attempted” [Mesh] OR suicid∗ OR self-harm OR depress∗). While the results from these studies are reassuring in the diabetic or obese populations, none of the currently published studies clearly addresses the issue of safety in patients with preexisting mental health disorders.

**Added value of this study**

This large nationwide case-time-control study provides reassuring information to clinicians and patients regarding the short-term psychiatric safety of GLP-1 RA. Especially, it complements the existing evidence by showing no specific risk for patients with history of psychiatric disorders or obesity.

**Implications of all the available evidence**

Our findings suggest no risk of suicide attempt or suicide with recent GLP-1 RA use, regardless of psychiatric history. This is particularly relevant for patients with psychiatric disorders, where some treatments, such as antipsychotics or antidepressants, are associated with weight gain, potentially making these patients suitable for GLP-1 RA treatment.

## Introduction

Glucagon-like peptide-1 receptor agonists (GLP-1 RA) are indicated for the treatment of type 2 diabetes and for weight loss (liraglutide and semaglutide). They stimulate insulin secretion, reduce glucagon secretion, delay gastric emptying, and act centrally to reduce appetite. As previous centrally-acting appetite suppressants were withdrawn due to a risk of suicidality, GLP-1 RA have come under particular scrutiny for psychiatric adverse events.^1^ Results from rodent models suggesting that acute activation of GLP-1 receptors was associated with anxiogenic effects, even though chronic activation was not and could conversely lead to a reduction in depressive behaviors, reinforced the need for such monitoring especially over the short-term.^2^^,^^3^

No psychiatric risk has been demonstrated in clinical trials, which have reported a few cases of depressive disorders, suicidal ideation and suicidal behaviors, but with no significant difference compared to placebo.^1^ A post-hoc analysis of four clinical trials on high-dose semaglutide in obesity provided consistent results.^4^ However, due to the pre-existing concerns about appetite-suppressants,^5^^,^^6^ patients with psychiatric history were largely excluded from these trials, limiting the generalizability of their findings to psychiatric populations.

Reassuring though it is, current real-life information does not entirely fill this gap. Some pharmacovigilance case reports and studies have raised concern, but the results are mostly inconsistent.^7^, ^8^, ^9^ Results from pharmaco-epidemiological studies are more homogeneous, at least in their direction; however none of the currently published studies clearly addresses the issue of safety in patients with preexisting mental health disorders.^10^, ^11^, ^12^, ^13^, ^14^ This appears an important gap, firstly given the increasing use of GLP-1 RA use in obese patients who are at increased risk of suicidality, and secondly given the potential of use of these drugs in patients treated with antipsychotics or antidepressants that can induce important weight gain.^15^^,^^16^ Recommendations from the agencies currently remained opposite. The FDA recommends close monitoring for mood changes, emerging or worsening depression, or suicidal behavior during treatment by the GLP-1 RA liraglutide and semaglutide. Conversely, the European Medicines Agency (EMA) has concluded that evidence is insufficient to support a causal association.^17^^,^^18^ This contrasting information might lead to situations of complex decision for clinicians, especially when envisioning prescribing GLP-1 RA in patients with risk factors for such events.

There is therefore an important need to assess the association between suicidal behaviors (suicide attempt and suicide death) and GLP-1 RA use according to psychiatric and obesity history.

## Methods

### Data source

A nationwide study was conducted from January 1, 2013 through December 31, 2021, using the French National Health Data System (SNDS). The SNDS contains sociodemographic and medical information on all outpatient services reimbursed by the National Health Insurance since 2006, including dispensed drugs, health expenditures for long-term chronic diseases and disabilities, hospital discharge summaries (including diagnoses and procedures) and causes of deaths. Indication and prescribed dosage are not directly informed in the database. Extensive description of the French medico-administrative databases is available elsewhere.^19^

### Ethics

By agreement of the French Data Protection Supervisory Authority (CNIL), neither ethics committee approval nor informed consent were required for this observational study based on anonymized French medico-administrative databases.

### Study design

We used a case-time-control (CTC) design, which is an extension of the case-crossover (CCO), to analyze the short-term risk of suicidal behaviors associated with GLP-1 RA use.^20^ Patients serving as their own control, this within-person comparison design enables the self-adjustment over a short period of time of individual confounding factors that vary slowly over time and are not generally recorded in medico-administrative databases, such as psychiatric symptoms or disorders (insomnia, depression, anxiety), substance or alcohol use, social isolation, financial difficulties, or family history of suicide. The CTC includes two CCO analyses: one in cases and one in a time-control group of non-diseased individuals. It was developed to address exposure-trend bias, a bias that could affect CCOs when prevalence of exposure vary in the general population over the study period as was the case for GLP-1 RA which use has considerably increased over the last year. In addition, the CTC controls for the persistent user bias associated with violation of the transient exposure assumption of the CCO when exposure is chronic.^21^^,^^22^ The CTC controls for these biases by correcting the CCO estimates in the cases using the corresponding CCO estimates in the time-controls.

### Suicide attempt and suicide death

A composite outcome for suicidal behaviors was defined, that combined hospitalization for a suicide attempt and death by suicide. If a patient experienced more than one event, only the first one was considered. Hospitalized suicide attempts were identified through the hospital discharge codes from the International Classification of Diseases, 10th revision (ICD-10; ×60–×84 for intentional self-harm). Suicides were identified through the causes-of-death registry as those with an ICD-10 codes ×60–×84 as well. While intentional self-harm does not equal to suicide attempt or suicide, the fact that only hospitalized acts or death are measured here suggest that most of them were considered severe and probably suicidal. The date of hospital admission (suicide attempt) or the date of death (suicide) constituted the index date. For individuals who died in hospital with a diagnostic code of suicide attempt, the outcome was considered a suicide but the date of admission served as the index date. This method of identifying suicide attempts and suicides in the SNDS database has been used in previous studies.^23^, ^24^, ^25^

### Exposure

The studied GLP-1 RA approved in France during the study period were exenatide, dulaglutide, liraglutide and semaglutide. These drugs can only be obtained through medical prescription, and their dispensing always leads to reimbursement, except for liraglutide medication specifically labeled for obesity (introduced on the French market in March 2021), whose dispensing was therefore not captured (details on codes provided in Supplementary Table S1).

**Fig. 1:**
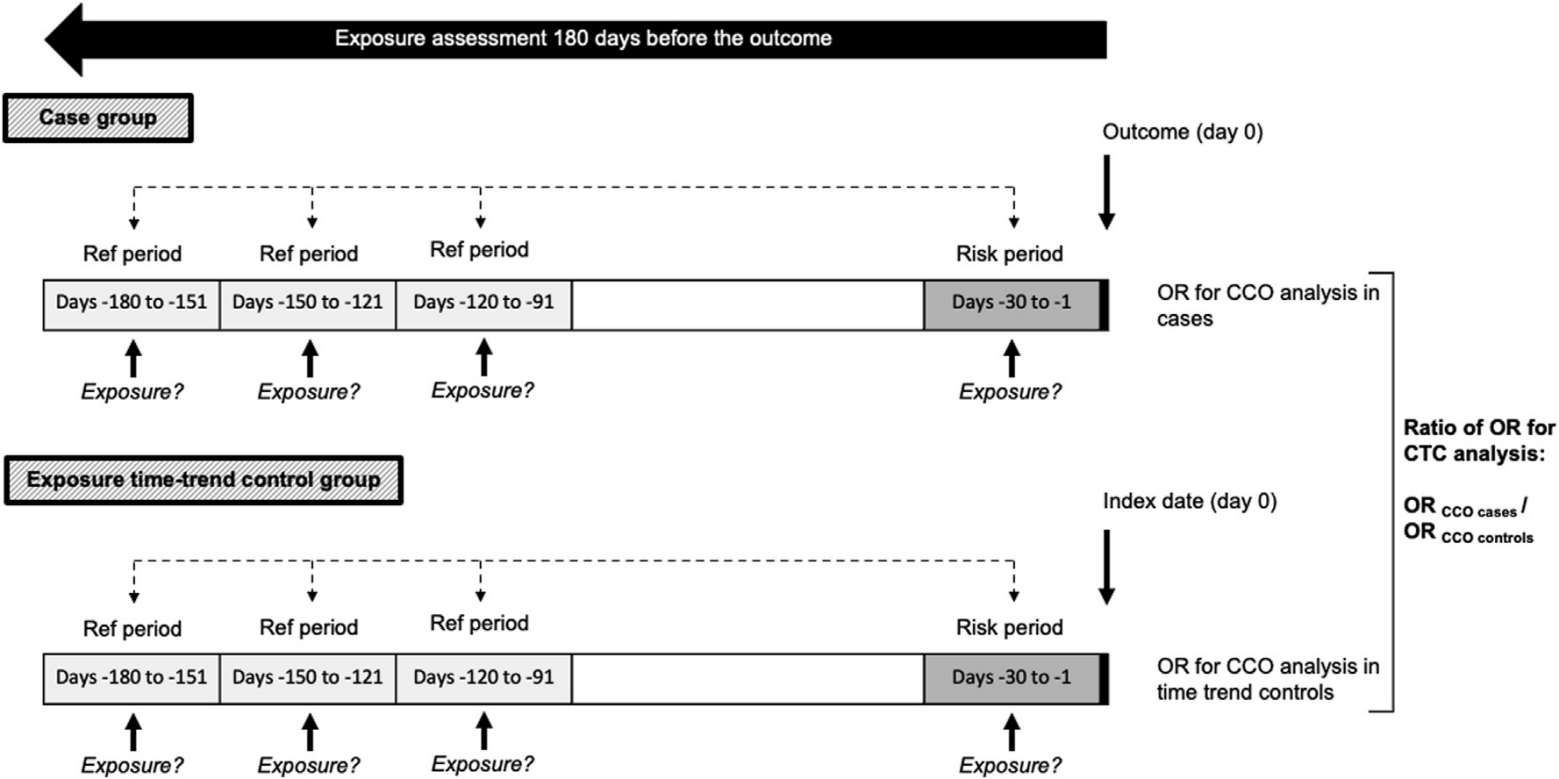
Diagram of the case-time-control design for studying the association between GLP-1 RA and suicidal behaviors. CCO, case crossover; CTC, case-time-control; OR, odds ratio; Ref period, reference period.

In the objective of assessing short-term risk, we considered 30-day periods for exposure assessment, this length also corresponding to the maximal time covered by one GLP-1 RA dispensing in France. Fig. 1 presents the CTC design and details on the exposure assessment periods: we identified a risk period of 30 days immediately preceding the suicidal act (days −30 to −1) and three reference periods of similar duration (days −180 to −151, −150 to −121, −120 to −91 before the suicidal act). A 60-day washout gap was defined between risk and reference periods. As conditionings for GLP-1 RA products available in France are thought to cover 30 days of treatment, this allowed considering that dispensings that would have been performed at the very late times of the closest reference window would be very unlikely to result in unidentified exposures in the risk one and that the washout length prevented any residual effect of exposure in the reference periods on the act. GLP-1 RA dispensed on the day of the act was not considered.

### Study population

The case group of the CTC analysis comprised all adults who met the following inclusion criteria: patients aged 18 and over who had been hospitalized for a suicide attempt or suicide between 1 January 2013 and 31 December 2021 (index date), and had at least one GLP-1 RA dispensing in the 180-day period preceding the index date (the “observation period”). Due to lack of information on in-hospital drug exposure in the database, patients hospitalized in the observation period were excluded from the study. The time-control group of the CTC included individuals who had used GLP-1 RA over the past 180 days and remained free of the outcome at the time a case occurred. Each case was matched with up to five controls by sex, birth year, presence or absence of at least one recent psychiatric history, and presence or absence of obesity, using risk-set sampling. A recent psychiatric history was defined as any hospitalization in a psychiatric department, long-term disease or disability for a psychiatric disorder, psychiatric consultation, or dispensing of psychotropics that occurred within the year preceding the observation period of exposure assessment. The identification of obesity relied on information from hospitalizations with obesity or bariatric surgery in the five years preceding the observation period (details on codes provided in Supplementary Table S1). For each individual case, the risk set sampling procedure randomly selects controls who match the selected criteria from all individuals in the source population who are free of the outcome of interest at the time the case experiences it. Time-controls were assigned an index date identical to that of the corresponding case.

### Statistics

Descriptive analyses were performed to characterize cases and time-controls at baseline. Data for qualitative variables are presented as number and proportion of patients in each group, and continuous variables as mean and standard deviation (SD).

We used conditional logistic models to estimate the crude and adjusted odds ratios (OR) of suicidal behaviors associated with GLP-1 RA over the risk period compared with the reference periods, for both cases and time-controls. The ratio between cases and time-controls OR provided an estimate controlling for exposure-trend and persistent-user biases. As CTC design is already self-adjusted for all time-stable confounders we only adjusted models for time-varying confounders: antidepressants, antipsychotics, anticonvulsants and lithium, which can exacerbate weight gain thus influencing GLP-1 RA use and might impact the risk of suicidal behaviors (details on codes provided in Supplementary Table S2).^26^ An individual was considered exposed for a period of interest (risk or reference) when a reimbursement for a dispensing occurred during this period. The analyses were stratified into four subgroups according to the existence of a recent psychiatric history and of obesity, the considering of these within the matching variables allowing performing this stratification without breaking the matching.

The CTC design cannot fully eliminate potential confounding by indication, which can occur if the reason for prescribing, as well as its severity, is time-depending and associated with an increased risk of the studied outcome. In the case of GLP-1 RA, if diabetes and overweight/obesity are chronic conditions, their seriousness or potential acute worsening is not and might lead to GLP-1 RA initiation. As it can also influence the risk of depression and suicidal behaviors, it might constitute time-dependent confounders.^15^^,^^27^ Another bias not inherently controlled by the CTC design is protopathic bias, whereby early symptoms of the disease condition the probability of exposure. In the case of suicidality occurring in patients with depressive symptomatology and potentially lesser search for specialized care such as endocrinology, it can lead to inverse associations for most non-psychotropic drugs.^24^ To evaluate the risk for such, we performed a bias analysis using, as negative control,^28^ dipeptidyl peptidase-4 (DPP-4) inhibitors, incretin mimetic drugs indicated for second-line treatment of diabetes with no effect and no pathophysiological rationale for suicide risk.^10^^,^^29^ The analysis considering DPP-4 inhibitors marketed in France (sitagliptin, vildagliptin, and saxagliptin) employed the same methods than the main analysis with GLP-1 RA.

Finally, we also conducted subgroup analyses according to sex and age (<60 years and ≥60 years) and sensitivity analyses to examine the robustness of our results using risk periods of 15 days and 45 days (reference periods adapted accordingly).

Data were analyzed using SAS Enterprise Guide® statistical software (SAS Institute, version 9.4, NC, USA).

### Role of funding source

The present study is part of the Drugs Systematized Assessment in real-liFe EnviRonment (Drugs-Safe®) centre research program that aims at providing an integrated system allowing the monitoring of drug use and safety in France. The potential impact of drugs, frailty of populations and seriousness of risks drive the research program. The Drugs-Safe® centre is funded by the French Medicines Agency (ANSM) (grant number 2024S001), in the context of a partnership with the Health Product Epidemiology Scientific Interest Group (EPI-PHARE). The ANSM played no role in the study design, conduct, and results interpretation or discussion. This publication represents the views of the authors and does not necessarily represent the opinion of the ANSM.

## Results

**Fig. 2:**
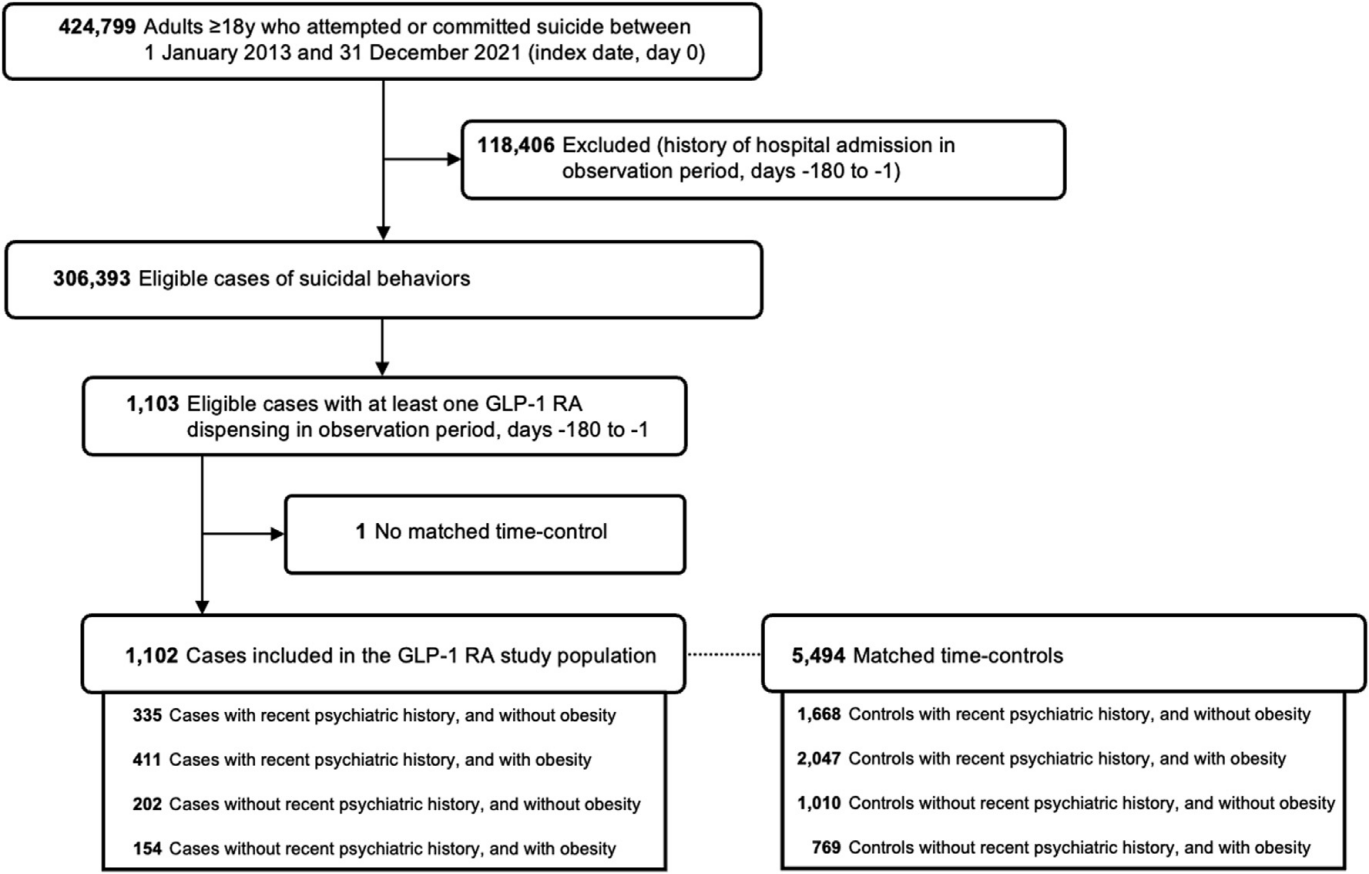
Flow-chart of eligible participants included in the GLP-1 RA study population.

From 2013 to 2021, 1102 patients who attempted or committed suicide and received GLP-1 RA during the observation period (180 days before suicidal act) fulfilled eligibility criteria and were matched to at least one time-control (Fig. 2).

Table 1 describes the baseline characteristics of the study population and the matched time-controls. Mean age of GLP-1 RA cases was 57.4 years (SD 11.2); 44.6% were male, 67.7% had a recent psychiatric history (including 59.2% with antidepressants use and 21.6% with antipsychotics use) and 51.3% had obesity. Regarding other comorbidities, 21.4% of GLP-1 RA cases had history of atherosclerotic cardiovascular disease, and 27.8% of sleep apnea. A more detailed description of the study population is presented in Supplementary Table S3.

**Table 1:** Descriptive characteristics of matched cases exposed to GLP-1 RA and their time-controls for the main analysis.

	GLP-1 RA users^a^	
	Cases N = 1102	Time-controls N = 5494
**Male**	492 (44.6)	2451 (44.6)
**Age (years)**	57.4 (11.2)	57.3 (11.3)
**Recent psychiatric history**^b^	746 (67.7)	3715 (67.6)
Stay in psychiatric department	57 (5.2)	108 (2.0)
Psychiatric consultation	24 (2.2)	104 (1.9)
LTD or disability for psychiatric disorder	404 (36.7)	1393 (25.4)
Affective disorder	296 (26.9)	912 (16.6)
Psychotic disorder	58 (5.1)	283 (5.2)
Psychoactive substance abuse	13 (1.2)	21 (0.4)
Other psychiatric illness	111 (10.1)	363 (6.6)
Psychotropic drugs dispensing	695 (63.1)	3299 (60.0)
Antidepressants	652 (59.2)	2950 (60.0)
Antipsychotics	238 (21.6)	812 (14.8)
Mood stabilizers, including lithium	101 (9.2)	265 (4.8)
**Cardiometabolic history**^c^		
Obesity	565 (51.3)	2816 (51.3)
Diabetes	1088 (98.7)	5431 (98.9)
Antidiabetic drugs dispensing		
GLP-1 RA	1000 (90.7)	4907 (89.3)
Biguanides	927 (84.1)	4835 (88.0)
Sulfonylureas	586 (53.2)	3166 (57.6)
Insulin	558 (50.6)	2606 (47.4)
DPP-4 inhibitors	366 (33.2)	2035 (37.0)
Repaglinide	201 (18.2)	892 (16.2)
Alpha glucosidase inhibitors	37 (3.4)	210 (3.8)
Thiazolidinediones	<10	20 (0.4)
ASCVD	236 (21.4)	969 (17.6)
Tobacco addiction treatment	145 (13.2)	474 (8.6)
Alcohol addiction treatment	42 (3.8)	74 (1.4)
Sleep apnea	306 (27.8)	1471 (26.8)

Data are n/N (%), or mean (SD). ASCVD, atherosclerotic cardiovascular disease; LTD, long-term disease.

a Cases and time-controls were matched by calendar time, sex, birth year, recent psychiatric history, and obesity.

b Recent psychiatric history in the year prior to the observation period (days −180 to −1).

c Cardiometabolic history in the 2 years prior to the observation period, until 5 years prior to this period for obesity identification.

**Fig. 3:**
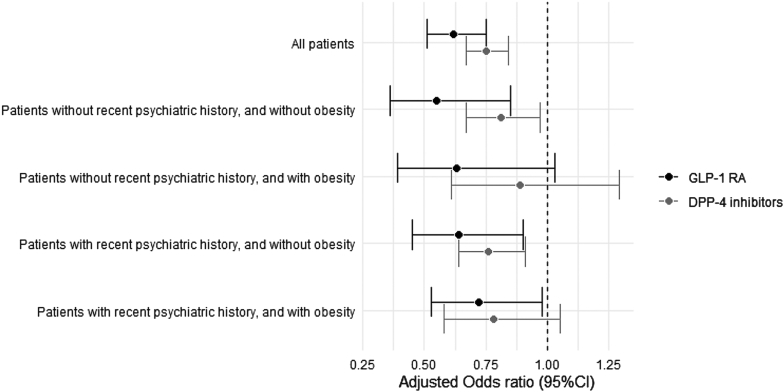
Association between suicidal behaviors and GLP-1 RA or DPP-4 inhibitors according to stratification on recent psychiatric history and/or obesity. Estimates presented are adjusted odds ratio with their 95% confidence interval (95% CI).

Of the 1102 cases exposed to GLP-1 RA, 780 were exposed in the risk period and 1045 in at least one reference period (Supplementary Figure S1). GLP-1 RA use was not associated with increased suicidal behaviors after adjustment for exposure trend and covariates (negative association: adjusted OR, 0.62; 95% CI, 0.51–0.75; Table 2). Results were similar whether patients had a recent psychiatric history or not, and whether they had or not history of obesity (Table 2 and Fig. 3).

**Table 2:** Crude and adjusted odds ratios for the risk of suicidal behaviors associated to GLP-1 RA use according to stratification on recent psychiatric history and/or obesity.

	Subjects	Antidiabetic use		Odds ratio (95% CI)		
		Risk period	Reference period^a^	Discordant pairs^b^	Crude	Adjusted
**All patients**						
CCO cases	1102	780	1045	379	0.89 (0.76–1.05)	0.81 (0.69–0.96)
CCO controls	5494	4220	5127	1641	1.29 (1.19–1.39)	1.31 (1.21–1.42)
CTC ratio					0.69 (0.58–0.83)	**0.62 (0.51**–**0.75)**
**Patients with recent psychiatric history, and without obesity**						
CCO cases	335	241	313	116	0.99 (0.73–1.33)	0.94 (0.68–1.28)
CCO controls	1668	1292	1531	513	1.42 (1.23–1.64)	1.47 (1.27–1.70)
CTC ratio					0.70 (0.50–0.97)	**0.64 (0.45**–**0.90)**
**Patients with recent psychiatric history, and with obesity**						
CCO cases	411	295	394	133	0.96 (0.74–1.24)	0.86 (0.65–1.14)
CCO controls	2047	1570	1941	583	1.17 (1.03–1.33)	1.20 (1.05–1.37)
CTC ratio					0.82 (0.61–1.09)	**0.72 (0.53**–**0.98)**
**Patients without recent psychiatric history, and without obesity**						
CCO cases	202	146	192	66	0.84 (0.57–1.23)	0.85 (0.57–1.26)
CCO controls	1010	783	921	316	1.55 (1.29–1.85)	1.56 (1.30–1.87)
CTC ratio					0.54 (0.36–0.83)	**0.55 (0.36**–**0.85)**
**Patients without recent psychiatric history, and with obesity**						
CCO cases	154	98	146	64	0.65 (0.43–1.00)	0.66 (0.43–1.03)
CCO controls	769	575	734	229	1.06 (0.86–1.29)	1.05 (0.86–1.29)
CTC ratio					0.62 (0.39–0.99)	**0.63 (0.39**–**1.03)**

CCO, case-crossover; CTC, case-time-control; CI, confidence interval.

Odds ratios were adjusted for time-varying confounders: antidepressants, antipsychotics, anticonvulsants, and lithium.

The adjusted CTC ratio is the final association estimate is indicated in bold.

a Individuals exposed in at least one reference period.

b Individuals either exposed in the risk or reference periods, yet not both.

Negative control analysis included 3615 DPP-4 inhibitor cases and 18,509 matched time-controls (Supplementary Figure S2). DPP-4 inhibitors cases, compared to GLP-1 RA cases, were older (mean age 62.5 years vs. 57.4 years, respectively), more often male (51.5% vs. 44.6%), had lesser history of recent psychiatric disorders (56.7% vs. 67.7%), and had lesser history of obesity (23.5% vs. 51.3%; Supplementary Table S3). Associations between DPP-4 inhibitor use and suicidal behaviors were similar to those obtained for GLP-1 RA, including after stratification according to the presence or absence of a recent psychiatric history and obesity (Fig. 3 and Supplementary Table S4).

Consistent results were obtained in analyses stratified on sex and age, and in sensitivity analyses using length of 15 and 45 days for the risk and reference periods (Supplementary Table S5 and Supplementary Table S6).

## Discussion

This French nationwide case-time-control study provides reassurance to clinicians and patients regarding the short-term psychiatric safety of GLP-1 RA overall and more specifically in obese patients and in patients with psychiatric disorders. The negative association found for GLP-1 RA (OR, 0.62; 95% CI, 0.51–0.75) was also found with our negative control DPP-4 inhibitors (OR, 0.75; 95% CI, 0.67–0.84). Consequently, we believe that reduced healthcare consumption (especially specialized care) in the period immediately preceding suicidal attempt is the more plausible explanation for the association found. A contraindication to the use of GLP-1 RA (i.e., treatment discontinuation) in patients with depression or suicidal ideation could be another explanation, but this could only be confirmed if the same effects were not found in the negative control group using DPP-4 inhibitors, which is not the case here. The findings remained robust across various subgroup and sensitivity analyses. This consistency strengthens the validity of our findings and suggests that the observed effect may be generalizable across diverse patient populations.

Our findings align overall with the existing real-world evidence regarding the psychiatric safety of GLP-1 RA and more specifically with that provided by other pharmaco-epidemiological studies. In a UK-based study using the Clinical Practice Research Datalink (CPRD), GLP-1 RA use was not associated to new-onset depression or self-inflicted injuries including suicide in comparison to sulfonylurea use (hazard ratio (HR), 1.25; 95% CI, 0.63–2.50) although these results may be limited by insufficient statistical power due to its small sample size (501 patients exposed to GLP-1 RA).^10^ A recent US-based cohort study utilized the TriNetX database to examine the risk of suicidal behaviors in a cohort of in 240,618 overweight or obese patients and 1,589,855 diabetic patients over a 6-month period. Comparing semaglutide to non-GLP-1 RA medications, results showed semaglutide was associated with lower risks of incident (HR, 0.27; 95% CI, 0.24–0.31) and recurrent (HR, 0.44; 95% CI, 0.32–0.60) suicidal ideation in overweight/obese patients, with similar findings in diabetic patients. These associations were consistent across sex, age, and ethnicity.^14^ A second US-based cohort study utilized the TriNetX database to examine the one-year risk of suicidal behaviors in a matched cohort of diabetic patients treated with semaglutide compared to sitagliptin, empagliflozin or glipizide. Results showed no association between semaglutide and depression, anxiety disorder or suicidality.^11^ A study commissioned by the EMA, utilizing the UK IQVIA database, found no significant increase in suicidal ideation and behaviors among GLP-1 RA users compared to sodium-glucose co-transporter 2 (SGLT2) inhibitor users (HR, 1.10; 95% CI, 0.86–1.41). However, contrary to our findings, the authors noted a slightly elevated risk in patients with both type 2 diabetes and obesity (HR, 1.33; 95% CI, 1.02–1.72).^30^ A target trial emulation using Medicare data investigated the risk of suicidal ideation and behaviors in older adults with type 2 diabetes associated with GLP-1 RA compared to SGLT2 inhibitors and DPP-4 inhibitors. The results showed no clear increased risk although the estimates were imprecise (GLP-1 RA vs. SGLT2 inhibitors: HR, 1.07; 95% CI, 0.80–1.45; GLP-1 RA vs. DPP-4 inhibitors; HR, 0.94; 95% CI, 0.71–1.24).^12^

Our study has several strengths that enhance the validity and generalizability of the findings. First, the French SNDS nationwide database provided a large, representative sample, enabling robust statistical analyses and strong external validity. Second, the case-time-control design offers a key methodological advantage, inherently adjusting for time-invariant confounders, both measured and unmeasured, which is crucial given the interplay between obesity, diabetes, and mental health. Including a time-control group further aids in controlling for temporal trends in medication use and suicidal behaviors. Third, our comprehensive identification of suicidal behaviors, encompassing both suicide attempts and suicides, provides a more complete picture of the association with GLP-1 RA than studies focusing solely on suicidal ideation. Finally, the consistency of findings across subgroups and sensitivity analyses strengthens the robustness and generalizability of the results.

However, our study has several limitations. First, this analysis based on medico-administrative databases lacked access to detailed clinical information. For example, we did not have granular data on the severity of diabetes or obesity, the intensity of depressive symptoms, or lifestyle factors. The case-time-control design we employed inherently controls for time-fixed confounders. To address time-varying confounding, we adjusted for factors such as the use of psychotropic drugs. However, residual confounding due to unmeasured time-varying factors (e.g., changes in BMI) cannot be entirely ruled out. By comparing exposure risk during different time periods within the same individual, the case-crossover design reduces the influence of unmeasured, stable variables like baseline BMI. Any residual confounding is likely limited to variations in these factors over the study's 6-month follow-up period. Such changes should be especially minimal for GLP-1 RA initiation that occurred shortly before an event of suicide or suicide attempt. Similarly, the potential influence of the duration of diabetes should be sharply reduced as limited to the potential influence of its increase in a subject over a 6-month period. Second, our study considered only the most severe cases of suicidal behaviors, namely hospitalized suicidal attempts and deaths by suicide, as we cannot identify suicidal attempts that did not result in hospitalization. This limits the generalizability of our results to less serious events of suicidality. Third, our study period spanned from 2013 to 2021, the last year we could have information for the cause of death. Even we could represent a large proportion of patients with obesity for which indications are more recent than for diabetes for instance, it is possible that the risk related to recent patterns of use in term of daily dosage could not be fully evaluated. Fourth, the precision of the association obtained from the smallest subgroup of cases studied, namely obese patients with no recent psychiatric history, was lower. However, this did not weaken the interpretation, as the estimate was almost identical to that obtained for the population as a whole. Finally, although our results are based on a large representative sample of the French population, generalization to other populations with different healthcare systems, prescribing practices, or genetic contexts may not be guaranteed.

While the results of our study did not identify any risk of suicidal behavior in the short-term following the use of GLP-1 RA, they do not allow us to assess a potential long-term risk. Further specifically designed research is necessary to confirm the long-term safety profile. Additionally, further investigation is needed to gain a deeper understanding of the risk in psychiatric subpopulations such as patients with schizophrenia. Concerns about the long-term psychiatric safety of GLP-1 RA are, however, currently limited to a few spontaneous post-marketing reports of depression or suicidal ideation with a long onset time. For physicians, the need for specific monitoring in psychiatric populations seems potentially more urgent, as GLP-1 RA may offer potential benefits in certain psychiatric conditions where treatments (e.g., antidepressants, antipsychotics) are associated with weight gain. This could result in an increase in their use in this indication. Research into these specific populations would require detailed clinical data, which are generally not available in most medico-administrative databases, such as the one used in our study.

This nationwide case-time-control study provides reassuring information to clinicians and patients regarding the short-term psychiatric safety of GLP-1 RA. Especially, it complements the existing evidence by showing no specific risk for patients with history of psychiatric disorders or obesity. Nonetheless, further research is needed to eliminate a potential long-term risk, and in specific at-risk population such as patients with schizophrenia.

## Contributors

All authors conceived and designed the study. EH and JB had access to and verified the underlying data. EH performed data management and conducted statistical analyses. JB and ABL ensured project and study management. JB, ABL, and JLF drafted the manuscript. All authors contributed to interpretation of the data and revised the manuscript. All authors read and approved the final version of the manuscript. JB and JLF are the guarantors. The corresponding author attests that all listed authors meet authorship criteria and that no others meeting the criteria have been omitted.

## Data sharing statement

No additional data available by author (French law to access SNDS https://www.snds.gouv.fr).

## Declaration of interests

All authors have completed the ICMJE uniform disclosure form and declare: all authors declare support from the French Medicines Agency (ANSM) for the submitted work. No other potential conflicts of interest relevant to this article were reported.
