# Supplementary material for: Suicide and suicide attempt in users of GLP-1 receptor agonists: a nationwide case-time-control study
Source: eClinicalMedicine. 2024 Dec 31;80:103029. doi: 10.1016/j.eclinm.2024.103029 (PMC11751538; doi:10.1016/j.eclinm.2024.103029)
Supplement: Supplementary file [file mmc1.docx]

**Supplementary material**

Supplementary Table 1. Codes for identification of exposure and characteristics at inclusion

Supplementary Table 2. Covariates for adjusted models

Supplementary Table 3. Complete descriptive characteristics of matched cases, exposed to GLP-1 RA or DPP-4 inhibitors, and their time-controls

Supplementary Table 4. Crude and adjusted odds ratios for the risk of suicidal behaviors associated with DPP-4 inhibitor use and according to stratification on recent psychiatric history and/or obesity

Supplementary Table 5. Results of main analysis stratified by sex and age. Crude and adjusted odds ratios for GLP-1RA use

Supplementary Table 6. Results of the sensitivity analyses for different risk periods. Crude and adjusted odds ratios for GLP-1 RA use

Supplementary Figure 1. Distribution of patients with use of GLP1-RA and DPP-4 inhibitors over the 180 days before suicide attempt or suicide

Supplementary Figure 2. Flow-chart of eligible participants included in the DPP-4 inhibitors study population

**Supplementary Table 1. Codes for identification of exposure and characteristics at inclusion**

|  | **Source** | **Codes** |
| --- | --- | --- |
| **Exposure of interest** |  |  |
| GLP-1 RA | Drug dispensing (ATC) | A10BJ01, A10BX04, A10BJ02, A10BX07, A10AE56, A10BJ05, A10BX14, A10BJ06 |
| DPP-4 inhibitors | Drug dispensing (ATC) | A10BH01, A10BD07, A10BH02, A10BD08, A10BH03, A10BD10 |
| **Characteristics at inclusion** |  |  |
| Recent psychiatric history |  |  |
| Stay in psychiatric department | Psychiatric hospital |  |
| Psychiatric consultation | French consultation codes | 1118, 2336, 1216, APY, AVY, CNP |
| LTD or disability for psychiatric disorder |  |  |
| Affective disorder | Registration for LTD or disability (ICD-10) | F30-F39 |
| Psychotic disorder | Registration for LTD or disability (ICD-10) | F20-F29 |
| Psychoactive substance abuse | Registration for LTD or disability (ICD-10) | F10-F19 |
| Other psychiatric illness | Registration for LTD or disability (ICD-10) | F40-43, F45, F50, F60, F62, F64-65, F69 |
| Psychotropic drugs dispensing |  |  |
| Antidepressants | Drug dispensing (ATC) | N06A |
| Antipsychotics | Drug dispensing (ATC) | N05A (excluding N05AN) |
| Mood stabilizers, including lithium | Drug dispensing (ATC) | N03AF01, N03AG02, N03AX09, N05AN |
|  | Drug dispensing (French classification) | 3400934876233, 3400935444271 |
| Obesity |  |  |
| Hospitalization with obesity | Hospital discharge diagnosis (ICD-10) | E66 |
| Bariatric surgery | Medical procedure (CCAM) | HFCA001, HFCC003, HFFA001, HFFA011, HFFC004, HFFC018, HFGC900, HFKA001, HFKA002, HFKC001, HFMA009, HFMA010, HFMA011, HFMC006, HFMC007, HFMC008, HGCA009, HGCC027 |
| ASCVD |  |  |
| Ischemic heart disease | Hospital discharge diagnosis (ICD-10) or registration for long-term disease (ICD-10) | I20-I25 |
| Stable angina | Drug dispensing (ATC) | C01DA, C01DX12, C01DX16 |
| Peripheral arterial disease | Hospital discharge diagnosis (ICD-10) or registration for long-term disease (ICD-10) | I70-I74 |
| Coronary revascularization | Medical procedure (CCAM) | DDAA002, DDAF001, DDAF003, DDAF004, DDAF006-DDAF010, DDFF001, DDFF002, DDPF002, DDMA002-DDMA009, DDMA011-DDMA013, DDMA015-DDMA038 |
| Ischemic stroke | Hospital discharge diagnosis (ICD-10) or registration for long-term disease (ICD-10) | I63 |
| Transient ischemic attack | Hospital discharge diagnosis (ICD-10) or registration for long-term disease (ICD-10) | G450, G451, G452, G453, G458, G459 |
| Diabetes, with or without complications | Hospital discharge diagnosis (ICD-10) or registration for long-term disease (ICD-10) | E10-E14  G590, G632, G730, G990, H280, H360, I792, L97, M142, M146, N083 |
|  | Drug dispensing (ATC) | A10A, A10B (excluding A10BX06, A10BJ, A10BK) |
| Sleep apnea | French medical device code | 1188684, 1130897, 1189991, 1162093, 1166688, 1188767, 1124460, 1118904, 1103156, 1126660, 1118614, 132608, 1187880, 1115455, 1192987, 1103446, 1162006, 1124112, 1106663, 1142417, 1152686, 1179248, 1129888, 1116911, 1154806, 1151250, 1102470, 2451474, 2412971, 2455325, 2497884, 2407378, 2462680 |
|  | Medical procedure (CCAM) | GLQP007, AMQP009 to AMQP015, LBLD017, YYYY465 |
| Tobacco-related diseases | Hospital discharge diagnosis (ICD-10) or registration for long-term disease (ICD-10) | F17, J41, J42, J43, J44, F17, T65.2, Z50.8, I73.1, Z58.7, Z71.6, Z72.0, J41, J42, J43, J44, J96.1 |
|  | Drug dispensing (ATC) | N07BA, N06AX12, R03BB05, R03BB04, R03BB07, R03BB06, R03AC18, R03AC19, R03AL03, R03AL04, R03AL05, R03AL06, R03AL08, R03AL09 |
| Alcohol-related diseases | Hospital discharge diagnosis (ICD-10) or registration for long-term disease (ICD-10) | F10, K70, T51, E24.4, F10, G31.2, G62.1, G72.1, I42.6, K29.2, K70, K86.0, T51, Z50.2, Z71.4, Z72.1, C70, C71, C79.3, C79.4, D32, D33, D42, D43, G04, G05, G06, G09, G12, G13, G24, G25, G26, G31, G32, G35, G36, G37, G46, G80, G81, G82, G83, G91, G93, G95 |
|  | Drug dispensing (ATC) | N07BB, M03BX01 |
|  | French biology code | 0519 |
|  | French medical device code | 4101353, 4164566, 4184899, 4169670, 4107723, 4118193, 4122473, 4179540, 4111854, 4147668, 4130136, 4122757, 4174323, 4159619, 4195615, 4142530, 4183434, 4168966, 4113920, 4252810, 4255116, 4263950, 4261051, 4233570, 4325302, 4371408, 4342654, 4327382, 4329040, 4324739, 4300348, 4321630, 4375613, 4375116, 4371555, 4359293, 4308597, 4339681, 4309674, 4307824, 4302152, 4389845, 4348622, 4307994, 4326431, 4371704 |

Abbreviations: ASCVD: atherosclerotic cardiovascular disease; ATC: Anatomical Therapeutic Chemical classification; CCAM: French classification for medical procedure; ICD-10: International Statistical Classification of Diseases and Related Health Problems 10th Revision; LTD: long-term disease

**Supplementary Table 2. Covariates for adjusted models**

| **ATC codes** | **Name** |
| --- | --- |
| N06A | Antidepressants |
| N05A, except N05AN | Antipsychotics, except lithium |
| N05AN | Lithium |
| N03A, except N03AX11 | Anticonvulsants, except topiramate |

**Supplementary Table 3. Complete descriptive characteristics of matched cases, exposed to GLP-1 RA or DPP-4 inhibitors, and their time-controls**

|  | | |  | **GLP-1 RA users^*^** | | **DPP-4 inhibitor users^*^** | |
| --- | --- | --- | --- | --- | --- | --- | --- |
|  | | |  | **Cases**  **N=1,102** | **Time-controls**  **N=5,494** | **Cases**  **N=3,615** | **Time-controls**  **N=18,059** |
| **Male** | | | | 492 (44·6) | 2,451 (44·6) | 1,861 (51·5) | 9,297 (51·5) |
| **Age (years)** | | | | 57·4 (11·2) | 57·3 (11·3) | 62·5 (12·5) | 62·5 (12·5) |
| **Social deprivation index**^†^ | | | |  |  |  |  |
|  | Quintile 1 (the least deprivation) | | | 92 (8·3) | 694 (12·6) | 375 (10·4) | 2,406 (13·3) |
|  | Quintile 2 | | | 160 (14·5) | 826 (15·0) | 511 (14·1) | 2,829 (15·7) |
|  | Quintile 3 | | | 187 (17·0) | 1,025 (18·7) | 669 (18·5) | 3,271 (18·1) |
|  | Quintile 4 | | | 267 (24·2) | 1,104 (20·1) | 798 (22·1) | 3,692 (20·4) |
|  | Quintile 5 (the most deprivation) | | | 325 (29·5) | 1,387 (25·2) | 1,049 (29·0) | 4,434 (24·6) |
|  | Unknown | | | 71 (6·4) | 458 (8·3) | 213 (5·9) | 1,427 (7·9) |
| **Index year** | | | |  |  |  |  |
|  | 2013 | | | 70 (6·4) | 350 (6·4) | 431 (11·9) | 2,153 (11·9) |
|  | 2014 | | | 99 (9·0) | 493 (9·0) | 407 (11·3) | 2,035 (11·3) |
|  | 2015 | | | 102 (9·3) | 506 (9·2) | 400 (11·1) | 2,000 (11·1) |
|  | 2016 | | | 100 (9·1) | 500 (9·1) | 436 (12·1) | 2,174 (12·0) |
|  | 2017 | | | 110 (10·0) | 548 (10·0) | 367 (10·2) | 1,832 (10·1) |
|  | 2018 | | | 126 (11·4) | 630 (11·5) | 424 (11·7) | 2,116 (11·7) |
|  | 2019 | | | 143 (13·0) | 714 (13·0) | 396 (11·0) | 1,980 (11·0) |
|  | 2020 | | | 160 (14·5) | 797 (14·5) | 379 (10·5) | 1,895 (10·5) |
|  | 2021 | | | 192 (17·4) | 956 (17·4) | 375 (10·4) | 1,874 (10·4) |
| **Recent psychiatric history**^‡^ | | | | 746 (67·7) | 3,715 (67·6) | 2,051 (56·7) | 10,239 (56·7) |
|  | Stay in psychiatric department | | | 57 (5·2) | 108 (2·0) | 186 (5·2) | 260 (1·4) |
|  | Psychiatric consultation | | | 24 (2·2) | 104 (1·9) | 62 (1·7) | 333 (1·8) |
|  | LTD or disability for psychiatric disorder | | | 404 (36·7) | 1,393 (25·4) | 1,043 (28·9) | 3,642 (20·2) |
|  |  | Affective disorder | | 296 (26·9) | 912 (16·6) | 666 (18·4) | 2,192 (12·1) |
|  |  | Psychotic disorder | | 58 (5·1) | 283 (5·2) | 204 (5·6) | 759 (4·2) |
|  |  | Psychoactive substance abuse | | 13 (1·2) | 21 (0·4) | 53 (1·5) | 132 (0·7) |
|  |  | Other psychiatric illness | | 111 (10·1) | 363 (6·6) | 283 (7·8) | 1,022 (5·7) |
|  | Psychotropic drugs dispensing | | | 695 (63·1) | 3,299 (60·0) | 1,919 (53·1) | 8,989 (49·8) |
|  |  | Antidepressants | | 652 (59·2) | 2,950 (60·0) | 1,741 (48·2) | 7,850 (43·5) |
|  |  | Antipsychotics | | 238 (21·6) | 812 (14·8) | 660 (18·3) | 2,089 (11·6) |
|  |  | Mood stabilizers, including lithium | | 101 (9·2) | 265 (4·8) | 269 (7·4) | 749 (4·2) |
| **Cardiometabolic history**^§^ | | | |  |  |  |  |
|  | Obesity | | | 565 (51·3) | 2,816 (51·3) | 849 (23·5) | 4,233 (23·4) |
|  | Diabetes | | | 1,088 (98·7) | 5,431 (98·9) | 3,547 (98·1) | 17,746 (98·3) |
|  | Antidiabetic drugs dispensing | | |  |  |  |  |
|  |  | GLP-1 RA | | 1,000 (90·7) | 4,907 (89·3) | 153 (4·2) | 811 (4·5) |
|  |  | Biguanides | | 927 (84·1) | 4,835 (88·0) | 2,265 (62·7) | 11,645 (64·5) |
|  |  | Sulfonylureas | | 586 (53·2) | 3,166 (57·6) | 1,621 (44·8) | 8,380 (46·4) |
|  |  | Insulin | | 558 (50·6) | 2,606 (47·4) | 689 (19·1) | 2,959 (16·4) |
|  |  | DPP-4 inhibitors | | 366 (33·2) | 2,035 (37·0) | 3,332 (92·2) | 16,599 (91·9) |
|  |  | Repaglinide | | 201 (18·2) | 892 (16·2) | 518 (14·3) | 2,208 (12·2) |
|  |  | Alpha glucosidase inhibitors | | 37 (3·4) | 210 (3·8) | 157 (4·3) | 786 (4·4) |
|  |  | Thiazolidinediones | | <10 | 20 (0·4) | 21 (0·6) | 115 (0·6) |
|  | ASCVD | | | 236 (21·4) | 969 (17·6) | 720 (19·9) | 2,976 (16·5) |
|  |  | Ischemic heart disease, including ACS | | 147 (13·3) | 640 (11·7) | 468 (13·0) | 1,919 (10·6) |
|  |  | Stable angina | | 84 (7·6) | 325 (5·9) | 258 (7·1) | 1,049 (5·8) |
|  |  | Peripheral arterial disease | | 53 (4·8) | 247 (4·5) | 176 (4·9) | 776 (4·3) |
|  |  | Coronary revascularization | | 24 (2·2) | 106 (1·9) | 72 (2·0) | 322 (1·8) |
|  |  | Ischemic stroke | | 13 (1·2) | 51 (0·9) | 37 (1·0) | 182 (1·0) |
|  |  | Transient ischemic attack | | <10 | 20 (0·4) | 16 (0·4) | 65 (0·4) |
|  | Lifestyle habits | | |  |  |  |  |
|  |  | Tobacco addiction treatment | | 145 (13·2) | 474 (8·6) | 328 (9·1) | 908 (5·0) |
|  |  | Alcohol addiction treatment | | 42 (3·8) | 74 (1·4) | 171 (4·7) | 328 (1·8) |
|  | Sleep apnea | | | 306 (27·8) | 1,471 (26·8) | 437 (12·1) | 2,384 (13·2) |

Data are n/N (%), or mean (SD). ACS: acute coronary syndrome. ASCVD: atherosclerotic cardiovascular disease. LTD: long-term disease

^*^ Cases and time-controls were matched by calendar time, sex, birth year, recent psychiatric history, and obesity.

^†^ Unknown for French overseas territories.

^‡^ Recent psychiatric history in the year prior to the observation period (days -180 to -1)

^§^ Cardiometabolic history in the 2 years prior to the observation period, until 5 years prior to this period for obesity identification.

**Supplementary Table 4. Crude and adjusted odds ratios for the risk of suicidal behaviors associated with DPP-4 inhibitor use and according to stratification on recent psychiatric history and/or obesity**

|  |  | **Subjects** | **Antidiabetic use** | | | **Odds ratio (95% CI)** | |
| --- | --- | --- | --- | --- | --- | --- | --- |
|  |  |  | **Risk period** | **Reference period**^*^ | **Discordant pairs**^†^ | **Crude** | **Adjusted** |
| **All patients** | | |  |  |  |  |  |
|  | CCO cases | 3,615 | 2,737 | 3,423 | 1,070 | 1·00 (0·91-1·10) | 0·90 (0·81-0·99) |
|  | CCO controls | 18,059 | 14,331 | 17,089 | 4,698 | 1·16 (1·10-1·21) | 1·19 (1·14-1·25) |
|  | CTC ratio |  |  |  |  | 0·86 (0·78-0·96) | **0·75 (0·67-0·84)** |
| **Patients with recent psychiatric history, and no obesity** | | | | | | | |
|  | CCO cases | 1,497 | 1,127 | 1,424 | 443 | 0·97 (0·83-1·12) | 0·93 (0·79-1·08) |
|  | CCO controls | 7,481 | 5,927 | 7,092 | 1,943 | 1·13 (1·06-1·22) | 1·22 (1·13-1·31) |
|  | CTC ratio |  |  |  |  | 0·85 (0·73-1·00) | **0·76 (0·64-0·91)** |
| **Patients with recent psychiatric history, and obesity** | | | | | | | |
|  | CCO cases | 554 | 423 | 531 | 154 | 1·02 (0·80-1·31) | 0·94 (0·72-1·22) |
|  | CCO controls | 2,758 | 2,175 | 2,609 | 732 | 1·12 (1·00-1·25) | 1·19 (1·05-1·35) |
|  | CTC ratio |  |  |  |  | 0·92 (0·70-1·20) | **0·78 (0·58-1·05)** |
| **Patients with no recent psychiatric history, and no obesity** | | | | | | | |
|  | CCO cases | 1,269 | 968 | 1,184 | 386 | 1·05 (0·89-1·24) | 0·99 (0·84-1·17) |
|  | CCO controls | 6,345 | 5,072 | 5,991 | 1,627 | 1·23 (1·14-1·33) | 1·22 (1·13-1·32) |
|  | CTC ratio |  |  |  |  | 0·86 (0·72-1·03) | **0·81 (0·67-0·97)** |
| **Patients with no recent psychiatric history, and obesity** | | | | | | | |
|  | CCO cases | 295 | 219 | 284 | 87 | 0·88 (0·63-1·22) | 0·94 (0·67-1·31) |
|  | CCO controls | 1,475 | 1,157 | 1,397 | 396 | 1·06 (0·80-1·23) | 1·06 (0·90-1·23) |
|  | CTC ratio |  |  |  |  | 0·84 (0·58-1·20) | **0·89 (0·61-1·29)** |

Abbreviations: CCO case-crossover; CTC case-time-control; CI confidence interval.

^*^ Individuals exposed in at least one reference period.

^†^ Individuals either exposed in the risk or reference periods, yet not both.

Odds ratios were adjusted for time-varying confounders: antidepressants, antipsychotics, anticonvulsants, and lithium, unless the number of subjects was less than 5.

**Supplementary Table 5. Results of main analysis stratified by sex and age. Crude and adjusted odds ratios for GLP-1 RA use**

|  | |  | **Antidiabetic use** | | | **Odds ratio (95%CI)** | |
| --- | --- | --- | --- | --- | --- | --- | --- |
|  | | **Subjects** | **Risk**  **period** | **Reference period**^*^ | **Discordant pairs**^†^ | **Crude** | **Adjusted** |
| **Sex** | |  |  |  |  |  |  |
| **Male** | |  |  |  |  |  |  |
|  | CCO cases | 492 | 347 | 468 | 169 | 0·82 (0·65-1·05) | 0·73 (0·57-0·94) |
|  | CCO controls | 2,451 | 1,926 | 2,265 | 711 | 1·42 (1·26-1·60) | 1·45 (1·29-1·64) |
|  | CTC ratio |  |  |  |  | 0·58 (0·44-0·76) | **0·50 (0·38-0·66)** |
| **Female** | |  |  |  |  |  |  |
|  | CCO cases | 610 | 433 | 577 | 210 | 0·95 (0·77-1·19) | 0·91 (0·72-1·14) |
|  | CCO controls | 3,043 | 2,294 | 2,862 | 930 | 1·20 (1·08-1·32) | 1·21 (1·09-1·34) |
|  | CTC ratio |  |  |  |  | 0·80 (0·63-1·01) | **0·75 (0·59-0·97)** |
| **Age, years** | |  |  |  |  |  |  |
| **<60** | |  |  |  |  |  |  |
|  | CCO cases | 631 | 433 | 591 | 238 | 0·95 (0·78-1·17) | 0·86 (0·70-1·07) |
|  | CCO controls | 3,139 | 2,348 | 2,897 | 1,033 | 1·32 (1·19-1·45) | 1·35 (1·22-1·50) |
|  | CTC ratio |  |  |  |  | 0·72 (0·58-0·91) | **0·64 (0·50-0·81)** |
| **≥60** | |  |  |  |  |  |  |
|  | CCO cases | 471 | 347 | 454 | 141 | 0·81 (0·62-1·04) | 0·75 (0·57-0·98) |
|  | CCO controls | 2,355 | 1,872 | 2,230 | 608 | 1·24 (1·10-1·41) | 1·24 (1·10-1·41) |
|  | CTC ratio |  |  |  |  | 0·65 (0·49-0·86) | **0·60 (0·45-0·81)** |

Abbreviations: CCO case-crossover; CTC case-time-control; CI confidence interval.

^*^ Individuals exposed in at least one reference period.

^†^ Individuals either exposed in the risk or reference periods, yet not both.

Odds ratios were adjusted for time-varying confounders: antidepressants, antipsychotics, anticonvulsants, and lithium, unless the number of subjects was less than 5.

**Supplementary Table 6. Results of the sensitivity analyses for different risk periods. Crude and adjusted odds ratios for GLP-1 RA use**

|  | |  | **Antidiabetic use** | | | **Odds ratio (95%CI)** | |
| --- | --- | --- | --- | --- | --- | --- | --- |
|  | | **Subjects** | **Risk**  **period** | **Reference period**^*^ | **Discordant pairs**^†^ | **Crude** | **Adjusted** |
| **15-day risk period**^‡^ | |  |  |  |  |  |  |
|  | CCO cases | 1,085 | 463 | 1,015 | 692 | 0·94 (0·83-1·07) | 0·87 (0·75-1·00) |
|  | CCO controls | 5,396 | 2,504 | 5,017 | 3,271 | 1·08 (1·02-1·15) | 1·07 (1·01-1·14) |
|  | CTC ratio |  |  |  |  | 0·87 (0·75-1·00) | **0·81 (0·69-0·95)** |
| **45-day risk period**^§^ | |  |  |  |  |  |  |
|  | CCO cases | 1,116 | 920 | 1,045 | 267 | 1·01 (0·83-1·24) | 0·98 (0·80-1·20) |
|  | CCO controls | 5,564 | 4,877 | 5,144 | 1,107 | 1·52 (1·38-1·69) | 1·56 (1·41-1·73) |
|  | CTC ratio |  |  |  |  | 0·66 (0·53-0·83) | **0·63 (0·50-0·79)** |

Abbreviations: CCO case-crossover; CTC case-time-control; CI confidence interval.

^*^ Individuals exposed in at least one reference period.

^†^ Individuals either exposed in the risk or reference periods, yet not both.

^‡^ Observation period: 135 days before the suicidal act. Risk period: days -15 to -1. Three matched reference periods: days -135 to -121, days -120 to -106, and days -105 to -91.

^§^ Observation period: 180 days before the suicidal act. Risk period: days -45 to -1. Two matched reference periods: days -180 to -136, and days -135 to -91.

Odds ratios were adjusted for time-varying confounders: antidepressants, antipsychotics, anticonvulsants, and lithium.

**Supplementary Figure 1 Distribution of patients with use of GLP1-RA and DPP-4 inhibitors over the 180 days before suicide attempt or suicide**

**
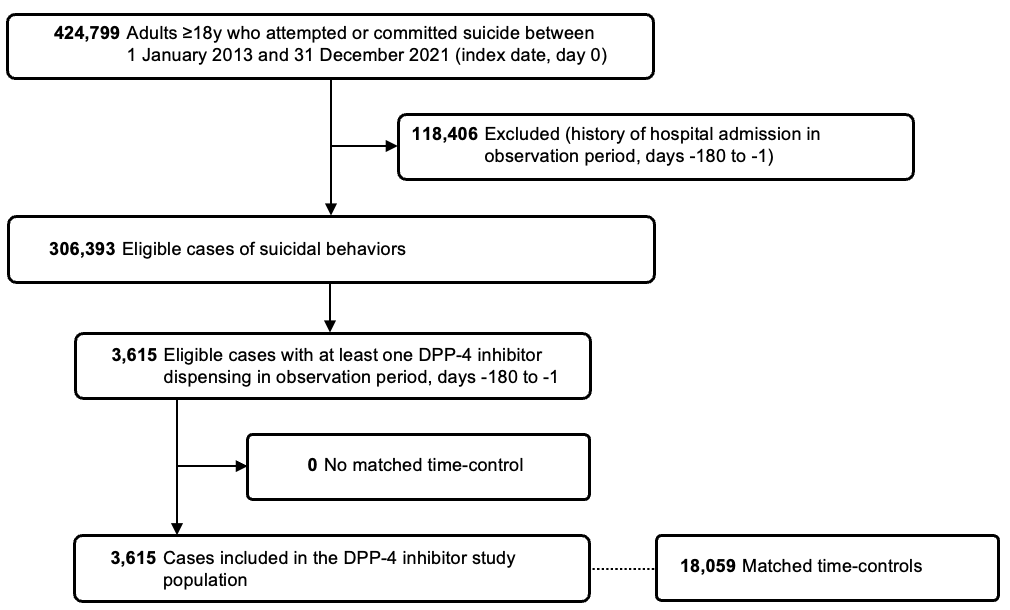
**

**Supplementary Figure 2 Flow-chart of eligible participants included in the DPP-4 inhibitors study population**
